# Different Oceanographic Regimes in the Vicinity of the Antarctic Peninsula Reflected in Benthic Nematode Communities

**DOI:** 10.1371/journal.pone.0137527

**Published:** 2015-09-10

**Authors:** Freija Hauquier, Laura Durán Suja, Julian Gutt, Gritta Veit-Köhler, Ann Vanreusel

**Affiliations:** 1 Marine Biology Research Group, Ghent University, Ghent, Belgium; 2 Alfred Wegener Institute, Helmholtz Centre for Polar and Marine Research, Bremerhaven, Germany; 3 Senckenberg am Meer, German Centre for Marine Biodiversity Research, Wilhelmshaven, Germany; Auckland University of Technology, NEW ZEALAND

## Abstract

Marine free-living nematode communities were studied at similar depths (~500 m) at two sides of the Antarctic Peninsula, characterised by different environmental and oceanographic conditions. At the Weddell Sea side, benthic communities are influenced by cold deep-water formation and seasonal sea-ice conditions, whereas the Drake Passage side experiences milder oceanic conditions and strong dynamics of the Antarctic Circumpolar Current. This resulted in different surface primary productivity, which contrasted with observed benthic pigment patterns and varied according to the area studied: chlorophyll *a* concentrations (as a proxy for primary production) were high in the Weddell Sea sediments, but low in the surface waters above; this pattern was reversed in the Drake Passage. Differences between areas were largely mirrored by the nematode communities: nematode densities peaked in Weddell stations and showed deeper vertical occurrence in the sediment, associated with deeper penetration of chlorophyll *a* and indicative of a strong bentho-pelagic coupling. Generic composition showed some similarities across both areas, though differences in the relative contribution of certain genera were noted, together with distinct community shifts with depth in the sediment at all locations.

## Introduction

The Antarctic Peninsula and surrounding Southern Ocean have been studied extensively during past decades due to their relevance in a historical, climatological, ecological and biogeographical context. Ever since the opening of the Drake Passage in the Oligocene (32–23 Ma; [1–2]; but see also [3]) and the subsequent establishment of the Antarctic Circumpolar Current (ACC; [4]), the Antarctic Peninsula has lost its direct connection to southernmost South America. Faunal links and gene flow, however, are still recognisable for some taxonomic groups [5–7] and many authors argue that the Scotia Arc islands continue to serve as a “stepping-stone” route towards ‘true’ Antarctic waters [8–10]. Throughout history, the ACC has effectively isolated Antarctica from sub-Antarctic influences (although it cannot be seen as an impermeable barrier; [11–15]). The resulting gradual cooling of Southern Ocean waters (due to a decrease in atmospheric CO_2_ and changes in ocean circulation; [3,16]) inhibited successful settlement and survival of some animal taxa (e.g., decapod crabs and teleost fish), whereas others flourished (e.g., peracarid crustaceans and echinoderms; [8,17–18]). Not surprisingly, it is mainly this difference in seabed temperatures between the cold Southern Ocean and warmer waters north of the polar front that defines the nature of Antarctic benthic assemblages (Clarke et al., 2009). Over the course of history, they have adapted to the prevailing conditions and are usually vulnerable to environmental change [19–20].

Currently, the Antarctic Peninsula is classified as one of the regions worldwide that is experiencing rapid atmospheric and oceanic warming [21–22], and as such is amongst the fastest warming and changing regions on Earth [23]. It should therefore come as no surprise that consequences (either direct or indirect) can already be observed in both physical and chemical properties of the marine environment (e.g., southward movement of ACC; [24]), ice-shelf and sea-ice dynamics (e.g., large-scale ice-shelf disintegration; [25]), and characteristics of the marine food web (e.g., shifts in phytoplankton communities; [26–28]). Seafloor-inhabiting communities near the Antarctic Peninsula are strongly dependent on bentho-pelagic coupling for their every-day life. Variable conditions in ice cover, temperatures, hydrographic dynamics and circulation patterns, and seasonality in primary productivity all interfere with each other and play a significant role in the functioning and structuring of the Antarctic ecosystem [28–30]. Even though food supply in Antarctic waters is highly seasonal, related transfer and input of organic matter to the sediment is able to sustain an abundant benthic community [31–36]. In this regard, the quantity and quality of phytodetritus deposition to the marine sediment largely define the success of benthic fauna [37–41]. At the same time, current dynamics and water-mass origin influence a variety of benthic processes, such as larval dispersion, transport of nutrients, oxygenation of the sediment (enhancing bacterial activity; [42–43]), and growth, recruitment and feeding strategy of local fauna [44]. All these parameters have shaped benthic communities over time and will continue to do so in the near future. Climate change has added an extra dimension of complexity that cannot be ignored; imminent changes in physical parameters, productivity regimes and seasonality as a result of continued warming in the Antarctic Peninsula region will undoubtedly influence benthic communities, but the consequences are barely understood [45]. Understanding the responses of the benthos to such climate-induced changes therefore requires as much information as possible on all levels of the food web.

To this end, the main goal of expedition ANT-XXIX/3 in 2013 [46] was to assess a variety of taxonomic groups in the Antarctic Peninsula region, sampling from the high-Antarctic Weddell area through the Bransfield Strait towards ACC-controlled waters north of the South Shetland Islands. This region marks the transition from cold Weddell Sea waters to warmer waters of the ACC [47–48]. The associated shift in seabed temperatures is largely mirrored by megabenthic communities, with a change from suspension-feeding hexactinellid sponge-dominated communities at Weddell Sea continental shelves to more motile echinoderm-dominated assemblages north of the South Shetland Islands in the Drake Passage [47,49]. Apparently, the physical properties of the ACC and Weddell Sea water masses dictate these differences in megafaunal composition and feeding mode, hence, a similar pattern can be expected for other benthic components [50]. Within this broader framework, this study will look at the smaller meiobenthos (32–1000 μm) at both sides of the peninsula to relate patterns in distribution and diversity to pelagic and oceanographic processes and dynamics. More specifically, focus will be on the free-living nematodes, a phylum with high ecological relevance. Nematodes are ubiquitous and have always been widespread around the world, even in the most extreme habitats, and are normally present in high abundance [51–57]. They show a strong correlation with biochemical conditions and characteristics of the sediment, which in turn are influenced by surface-water dynamics. Additionally, the link between surface primary productivity and nematode community structure has been verified on different occasions [58–60], proving their dependence upon food input from photic layers.

In accordance with the findings for the megabenthos [47] in the area and with findings for other Southern Ocean nematode communities [58–60], it is hypothesized that:

1. regions with high surface primary production will support high nematode densities due to strong bentho-pelagic coupling,
2. nematode community structure will depend on the physical characteristics (mainly temperature, cfr. [50]) of the different water masses,
3. nematode genus composition and feeding mode will differ between both sides (cfr. pronounced shift in feeding mode, hence composition, of the surface-dwelling megafauna [47]).

## Materials and Methods

### Sampling area and strategy

**Fig 1:**
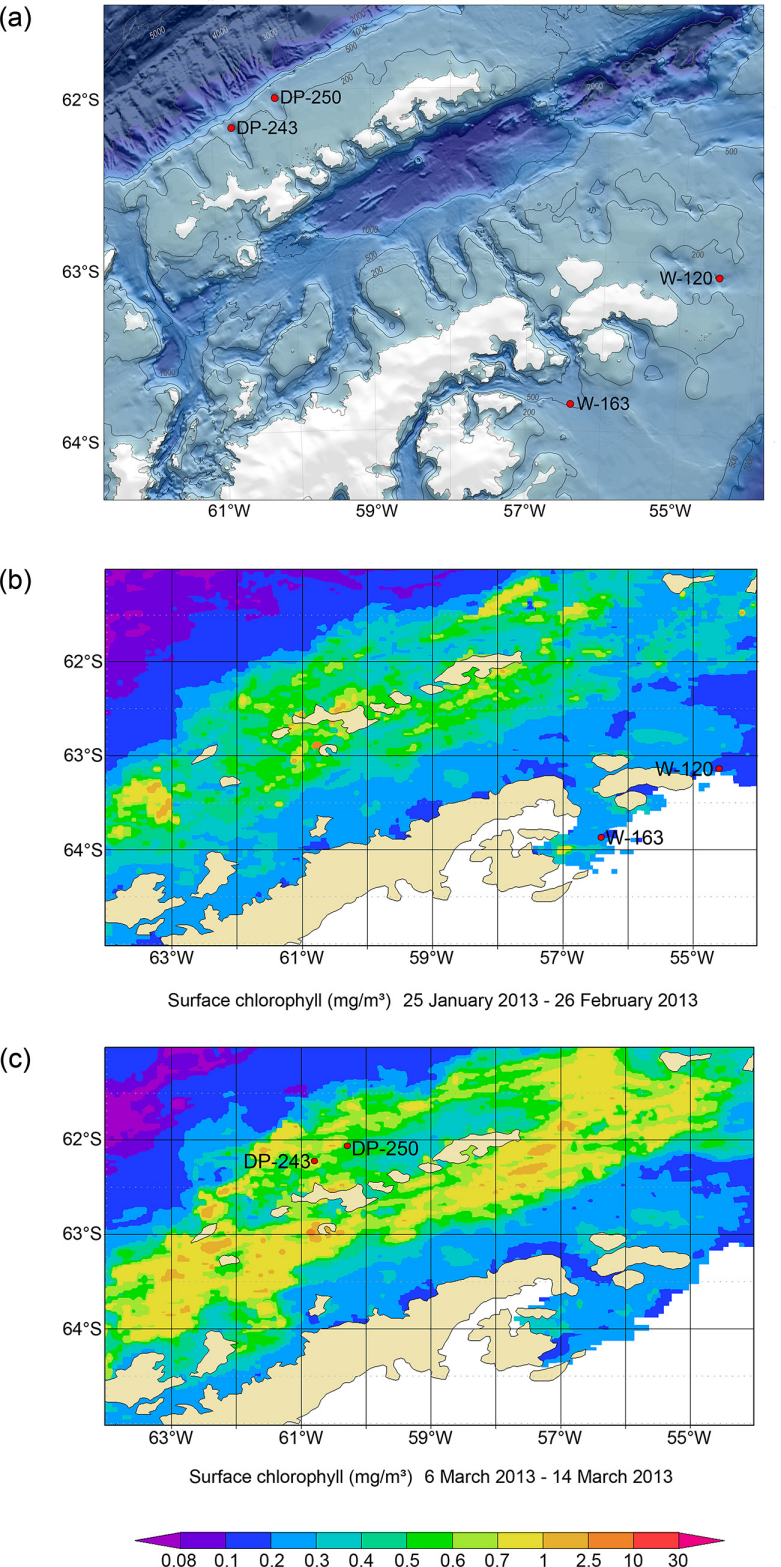
(a) Location of the four sampling stations (W-120 and W-163 east of the Antarctic Peninsula; DP-243 and DP-250 west in Drake Passage); map adapted from Alfred Wegener Institute bathymetry group; (b) + (c) Surface chl *a* concentrations (in mg m^-3^) at the respective sampling times for both sites. Graphs are based on MODIS Aqua data (NASA) of the sea surface on 8-day averages during the period of sampling and produced with the Giovanni online data system, developed and maintained by the NASA GES DISC.

Sampling was conducted near the Antarctic Peninsula during expedition ANT-XXIX/3 of the German icebreaking RV *Polarstern* in January–March 2013 [46], under permission of German (German Federal Environment Agency—Umweltbundesamt) and Belgian (Federal Public Service Health, Food Chain Safety and Environment—DG Environment) authorities, in compliance with the Antarctic Treaty System for all locations. No endangered or protected species have been collected for this study. Samples were taken at deep shelf depths (approx. 500 m) at two main locations: (1) northeast of the AP under Weddell Sea influence and (2) west of the AP, on the shelf of the South Shetland Islands in Drake Passage waters (Table 1, Fig 1A). Each location is represented by two stations with one CTD and three repeated multicorer (MUC) deployments (core diameter 57 mm; [61]). For clarity and consistency throughout this manuscript, the four stations will be abbreviated by using their location initials (W for Weddell; DP for Drake Passage) combined with the station number (e.g., 120).

**Table 1:** Details of the four sampling areas: each station was sampled once with a CTD, followed by three replicate MUC deployments.

Station	Gear	Replicate	Date	Latitude	Longitude	Depth (m)
**W-120**	CTD	1	28/01/2013	63°4.62´S	54°33.11´W	530.4
	MUC	1	28/01/2013	63°4.58´S	54°31.00´W	503.6
	MUC	2	28/01/2013	63°4.10´S	54°30.86´W	484.8
	MUC	3	28/01/2013	63°3.72´S	54°30.87´W	436.8
**W-163**	CTD	1	10/02/2013	63°53.07´S	56°26.19´W	468
	MUC	1	11/02/2013	63°50.95´S	56°24.43´W	517.6
	MUC	2	11/02/2013	63°51.01´S	56°23.97´W	516.6
	MUC	3	11/02/2013	63°51.03´S	56°23.68´W	517.1
**DP-243**	CTD	1	10/03/2013	62°12.27´S	60°44.42´W	497.4
	MUC	1	10/03/2013	62°12.32´S	60°44.47´W	497.8
	MUC	2	10/03/2013	62°12.31´S	60°44.48´W	497.7
	MUC	3	10/03/2013	62°12.31´S	60°44.54´W	495.2
**DP-250**	CTD	1	12/03/2013	62°2.28´S	60°12.11´W	487
	MUC	1	12/03/2013	62°2.22´S	60°12.01´W	489
	MUC	2	12/03/2013	62°2.24´S	60°12.06´W	488
	MUC	3	12/03/2013	62°2.24´S	60°12.03´W	488

One core from each replicate deployment was sliced per centimetre down to 5 cm depth and stored in a 4% formaldehyde-seawater solution for faunal analysis, while a second set of cores was collected for the analysis of environmental variables. These latter cores were sub-sampled with cut-off 10 mL syringes pushed into the sediment (0–5 cm) and stored at -20°C (granulometry, total organic carbon TOC and nitrogen TN) or -80°C (pigment content). In conjunction with sediment sampling, Niskin bottles mounted on a CTD rosette were deployed at chlorophyll-maximum (~20–50 m, defined by looking at in-situ chlorophyll profiles) and bottom depths (~450–510 m) of each station to assess water-mass properties (temperature and salinity) and chlorophyll content in the water column (see Fig 1B and 1C for surface chl *a* concentrations based on satellite data from NASA MODIS). Collected water was first poured over a 100 μm mesh to remove larger particles, after which 3 to 5 L was filtered at approximately 250 mbar over glass microfiber GF/C filters (1.2 μm pore size; [62]; no replication) until colouring of the filters became apparent. Filters were then stored at -80°C.

### Meiofauna and Nematoda

The upper 5 cm of the cores for faunal analysis were divided into cm-layers. Meiofauna was extracted from the sediment using two stacked sieves (upper limit 1 mm, lower limit 32 μm; [63]) and density gradient centrifugation (3 × 12 min at 3000 rpm) with Ludox HS-40 as a flotation medium (specific density of 1.18 g cm^-3^; [52,64]). All taxa were counted and identified under a stereomicroscope (magnification 50 ×) using the identification key of [65]. From each layer, 150 nematodes (all if the layer contained less than 150 individuals) were randomly selected, stored in anhydrous glycerol and mounted on glycerine slides for identification [66]. Genus-level identification (9000 specimens) was done with a Leica DMLS compound microscope (magnification 1000 ×), using the pictorial key to nematode genera of [67–68], the Nematoda chapter in the Handbook of Zoology [69] and the NeMys database [70]. Supplementary data on nematode genus composition is available at http://doi.pangaea.de/10.1594/PANGAEA.846306.

### Environmental characterisation

Chl *a* concentration in the water column (at both chlorophyll maximum and bottom depth) was determined with a fluorimeter from the GF/C filters. Concentrations are reported in μg L^-1^ (= equivalent to mg m^-3^). Pigment content of the sediment was measured with a fluorescence detector after separation using HPLC (High Performance Liquid Chromatography). Prior to analysis, syringe cores were divided at the same vertical resolution as faunal samples. Pigments were extracted from the lyophilised sediments by adding 10 mL of 90% acetone. For each slice, both chl *a* and phaeopigments (i.e. degradation products of chl *a*) were determined and results expressed in μg g^-1^. Chloroplastic pigment equivalents (CPE) are then the sum of chl *a* and phaeopigments, whereas their ratio indicates the amount of fresh material. Grain size was determined with laser diffraction (Malvern Mastersizer 2000, size range: 0.02–2000 μm) and size fractions were classified according to [71]. For simplicity reasons, fractions have been summed to restrict their number to three: silt+clay % (<63 μm), sand % (63–500 μm) and coarse sand % (>500 μm). Finally, percentages of total organic carbon (TOC) and nitrogen (TN) were determined by combustion on freeze-dried samples using a Flash 2000 organic elemental analyser (protocol available through Interscience B.V., Breda, The Netherlands; methodology similar to [72]). The ratio of C:N was calculated, multiplying with a factor 14:12 to account for the difference in molar mass of both elements.

### Data analysis

Faunal data were analysed using PRIMER v6 software [73] with the PERMANOVA+ add-on package [74]. Nematode genus data were standardised to individuals per 10 cm^2^ and square-root transformed to down-weight the importance of dominant genera prior to statistical analyses. Differences in community composition between stations and cm-layers were visualised using nMDS (non-metric multidimensional scaling) and CLUSTER based on a Bray-Curtis similarity matrix. Two-way crossed ANOSIM (Analysis of Similarities; factors ‘area’ = Weddell Sea or Drake Passage; and ‘layer’ = sediment depth; 9999 permutations) and SIMPER (Similarities of Percentages) quantified within- and between-station differences in community composition and contribution of genera to observed differences, respectively. PERMANOVA (permutational ANOVA) with four factors (‘area’ = fixed, ‘station’ = fixed, ‘layer’ = fixed, ‘replicate’ = random and nested within station; 9999 permutations) analysed differences in assemblages between stations and layers. Pairwise tests were performed between all pairs of levels for the different factors. True permutational p-values P(perm) were interpreted when the number of unique permutations exceeded 100, and Monte Carlo P-values P(MC) when this was not the case. PERMDISP tested for homogeneity of dispersions in the multivariate space of the different groups of significant factors (distances to centroids; p-value by permutation of least-squares residuals).

Draftsman plots were constructed for the environmental variables to check for skewness in data and for multi-collinearity. This resulted in a log-transformation for ‘median grain size’, and omission of variables ‘coarse sand %’, ‘chl *a*’, ‘phaeopigments’ and ‘TN’ (correlation >0.88 with others). A PCA plot was constructed based on the normalised values for all cm-layers and replicates of each station to look at variations in environmental setting between areas.

PRIMER software was also used to evaluate taxonomic diversity (N_0_ = number of genera; H’ = Shannon index (log*e*); EG(200) = expected number of genera in a sample of 200 individuals; Hill’s N_1_) and evenness (Hill’s N_inf_; J’ = Pielou’s evenness; see [75] and references therein). Functional diversity and trophic structure was approached by classifying nematode genera into feeding guilds according to the marine feeding type classification of [76]. Four different feeding types are recognised: selective (1A) and non-selective (1B) deposit feeders, epigrowth-feeders (2A) and omnivores/predators (2B). Based on this classification, one can calculate the trophic diversity index for each station (ITD): ITD = Σθ^2^ where θ is the contribution of each trophic group to total nematode density [75,77]. ITD ranges from 0.25 (highest trophic diversity, i.e. the four trophic guilds account for 25% each) to 1.0 (lowest diversity, i.e. one trophic guild accounts for 100% of total density). In this study, the inverse ITD^-1^ is used, ranging from 1 (low functional diversity) to 4 (high functional diversity). All biodiversity indices and feeding types were analysed and compared using one-way ANOVA as well as post-hoc pairwise comparisons between stations with R [78].

## Results

### Environmental characterisation

Satellite data of the area, averaged for the period of sampling (Fig 1B and 1C), showed a higher surface concentration of chl *a* in Drake Passage (0.5–0.7 mg m^-3^) than in the Weddell Sea (0.1–0.2 mg m^-3^). Unfortunately, severe seasonal sea-ice conditions and cloud cover prevented useful data acquisition in the months before and after sampling. Nevertheless, satellite observations were confirmed by surface water measurements (Table 2). On the contrary, bottom water measurements showed an opposite trend: concentrations were below detection limit in the Drake Passage whereas in the Weddell Sea they were <0.03 μg L^-1^. Other CTD measurements showed negligible variations in salinity between stations, yet lower temperatures in the Weddell Sea than in Drake Passage (Table 2).

**Table 2:** Water column properties at chl *a* max and bottom depth. Temperature and salinity are derived from CTD recordings, chl *a* from laboratory measurements.

		Temperature (°C)	Salinity (psu)	Chl *a* (mg m^-3^)
**W-120**	**Chl *a* max**	-1.81	34.31	0.088
	**Bottom**	-1.81	34.50	0.025
**W-163**	**Chl *a* max**	-1.48	34.30	0.070
	**Bottom**	-1.77	34.50	0.013
**DP-243**	**Chl *a* max**	1.19	34.20	0.589
	**Bottom**	0.99	34.60	0.000
**DP-250**	**Chl *a* max**	1.12	34.15	0.452
	**Bottom**	0.57	34.58	0.000

**Fig 2:**
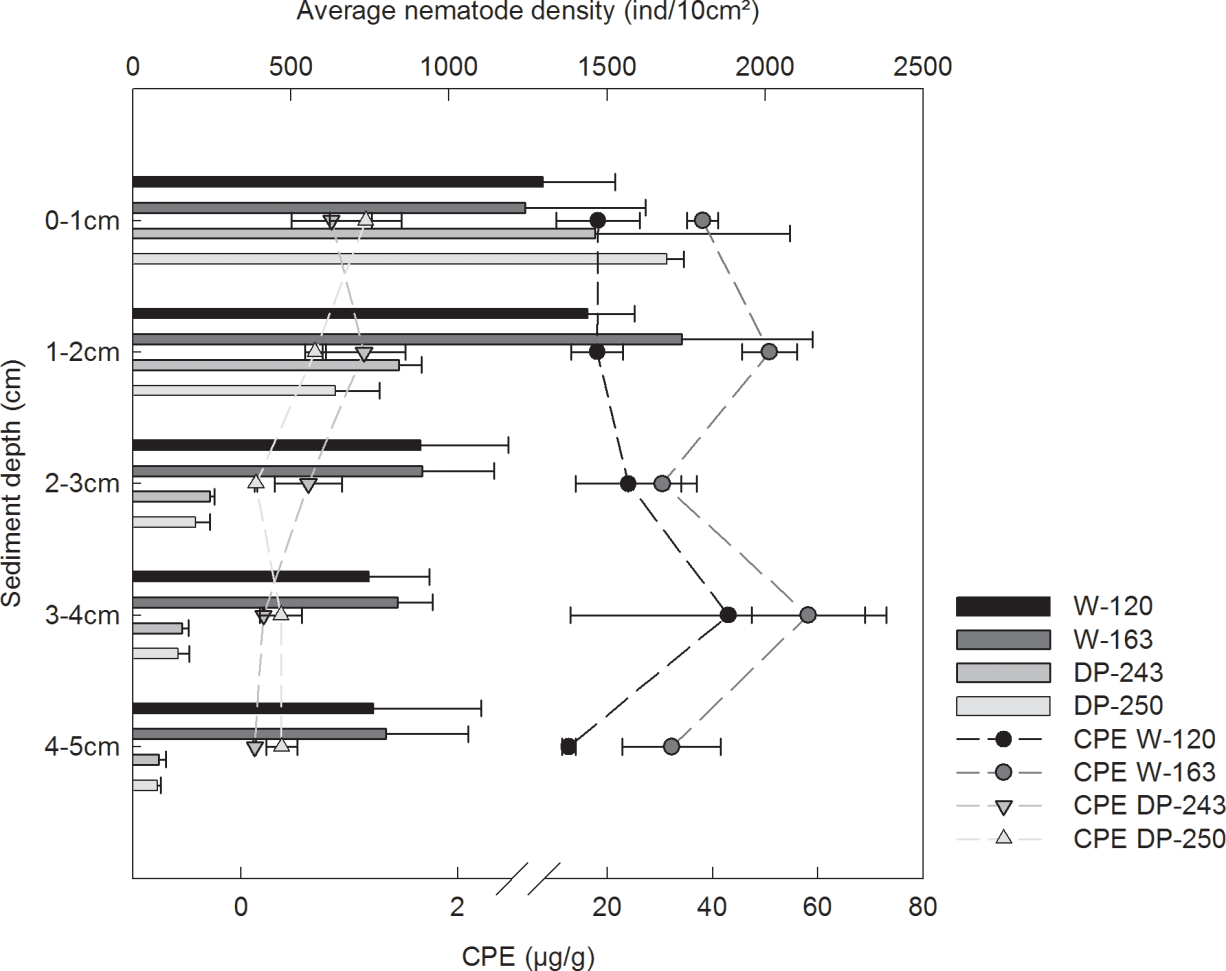
Vertical distribution of pigments and nematodes. Average CPE values (μg g^-1^; dots) and nematode densities (ind 10 cm^-2^, bars) with their respective standard error in the sediment for all four stations (n = 3).

**Fig 3:**
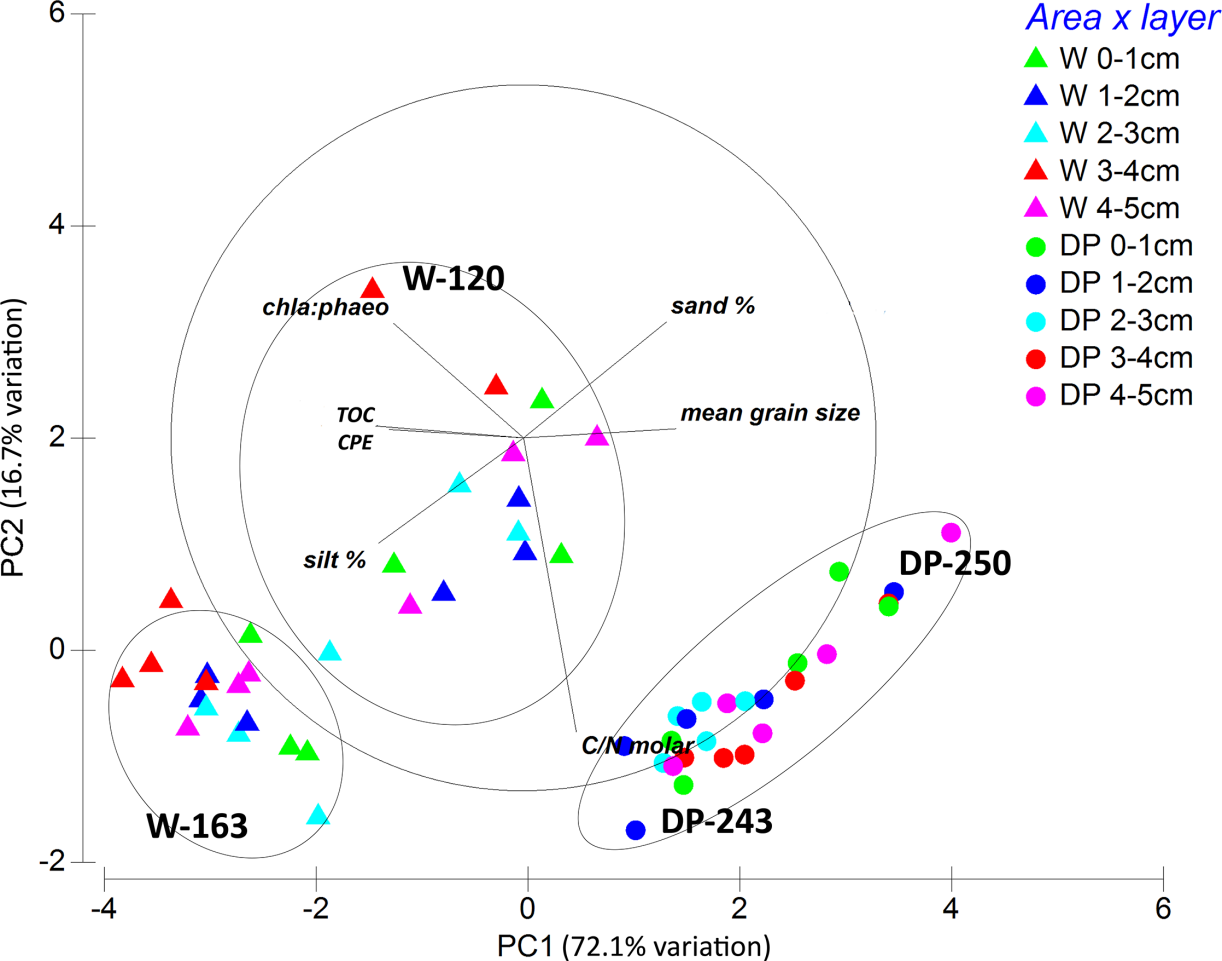
PCA plot of environmental data. Each symbol corresponds to a centimetre layer of a different replicate in Weddell Sea or Drake Passage and represents the environmental setting for that sample.

Within the sediment, mainly pigment values showed large differences between the two regions. Chl *a* and phaeopigment content was up to more than 100 times higher in Weddell Sea stations (Table 3), resulting in higher CPE concentrations and a higher amount of fresh material compared to Drake Passage. Vertical distribution of CPE values was different, too: while CPE concentrations remained high throughout the sediment depth layers in the Weddell Sea, their values decreased with each centimetre in the Drake Passage (Fig 2). Also TOC content peaked in the Weddell Sea (mainly W-163). On the contrary, DP-243 and DP-250 were characterised by coarser sediment than W-120 and W-163. Highest C:N_molar_ values were obtained in Drake Passage station DP-243. A PCA plot of the different stations (Fig 3) indicated that the environmental setting at the two Drake Passage stations was quite similar (stations DP-250 and DP-243 placed closer together), while there was a higher discrepancy in the case of Weddell Sea stations (but they are also more geographically separated than Drake Passage stations).

**Table 3:** Sedimentary environmental variables per station (± standard deviation), both for the upper centimetre separately and averaged over all replicates and layers.

	W-120		W-163		DP-243		DP-250	
	0–1 cm	0–5 cm	0–1 cm	0–5 cm	0–1 cm	0–5 cm	0–1 cm	0–5 cm
**Chl *a* (μg g** ^**-1**^ **)**	9.31 (7.57)	15.33 (10.23)	25.20 (4.81)	30.68 (11.11)	0.15 (0.13)	0.06 (0.06)	0.10 (0.03)	0.06 (0.03)
**Phaeo (μg g** ^**-1**^ **)**	8.96 (6.36)	7.89 (2.09)	12.92 (0.43)	11.27 (1.83)	0.69(0.39)	0.52 (0.38)	1.05 (0.55)	0.49 (0.37)
**CPE (μg g** ^**-1**^ **)**	18.27 (13.78)	23.22 (11.76)	38.12 (5.15)	41.96 (12.07)	0.83 (0.52)	0.58 (0.42)	1.15 (0.57)	0.54 (0.39)
**Chl *a*:phaeo**	1.04 (0.44)	1.94 (0.84)	1.95 (0.33)	2.72 (0.84)	0.22 (0.08)	0.11 (0.06)	0.09 (0.06)	0.11 (0.06)
**MGS (μm)**	37.58 (5.85)	35.56 (2.66)	27.10 (0.54)	24.79 (1.58)	49.81* (na)	48.75 (3.18)	78.43 (2.24)	69.24 (6.93)
**Silt+clay %**	83.96 (3.83)	84.61 (1.11)	91.94 (0.63)	93.74 (1.17)	84.07* (na)	84.28 (1.56)	73.46 (2.50)	76.73 (3.32)
**Sand %**	15.75 (3.75)	15.22 (1.08)	8.06 (0.63)	6.26 (1.17)	15.38* (na)	15.12 (1.55)	24.93 (3.02)	22.01 (3.38)
**Coarse sand %**	0.29 (0.50)	0.17 (0.23)	0.00 (0.00)	0.00 (0.00)	0.55* (na)	0.60 (0.04)	1.61 (0.53)	1.25 (0.30)
**TN %**	0.22 (0.01)	0.21 (0.02)	0.25 (0.02)	0.24 (0.01)	0.08 (0.00)	0.07 (0.00)	0.09 (0.02)	0.08 (0.01)
**TOC %**	1.13 (0.11)	1.09 (0.06)	1.64 (0.13)	1.56 (0.05)	0.56 (0.00)	0.52 (0.04)	0.61 (0.14)	0.53 (0.06)
**C:N**_**molar**_	6.00 (0.50)	6.18 (0.32)	7.54 (0.54)	7.51 (0.20)	8.29 (0.25)	8.18 (0.14)	7.76 (0.25)	7.97 (0.15)

(CPE = chloroplastic pigment equivalents; MGS = median grain size; silt+clay % = fraction <63 μm; sand % = between 63–500 μm; coarse sand % = fraction >500 μm; TN = % total nitrogen; TOC = % total organic carbon; C:N_molar_ = ratio of TOC:TN). *Values based on only one replicate measurement, due to large bias in data (i.e. stone present in 0–1 cm of replicate 243–3).

### Meiofauna and nematode abundance

Total meiofauna densities (averaged for the three replicates of each station) were twice as high in W-120 and W-163 (6235 ± 704 and 7196 ± 1274 ind 10 cm^-2^, respectively) than in DP-243 (3075 ± 1083 ind 10cm^-2^) and DP-250 (3049 ± 41 ind 10 cm^-2^). There was a significant difference in the number of individuals between Weddell Sea and Drake Passage (one-way ANOVA, p < 0.05) but not between stations of the same area (post-hoc pairwise comparisons). A total of 20 different taxa could be distinguished in the samples, with a clear dominance of nematodes in all samples of all four stations (average contribution 75–96% of total abundance). Nematodes were followed by harpacticoid copepods (1–13%), nauplius larvae (1–14%) and polychaetes (0.3–1.6%), after which a variety of other taxa was recognised in low numbers (e.g., Ostracoda, Kinorhyncha and Gastrotricha). Averaged nematode densities ranged between 2751 ± 82 (station DP-250) and 5532 ± 878 ind 10 cm^-2^ (station W-163). As for meiofauna, densities were higher in Weddell Sea stations than in Drake Passage. However, nematode density was similar in the first sediment layers (0–1 cm) of stations at both sides (approx. 1200–1700 ind 10 cm^-2^), and only started to vary from the second centimetre onwards. There was a steep decline in numbers with depth for Drake Passage stations, while Weddell Sea nematodes continued to be present in higher numbers even in the deeper layers (Fig 2).

### Nematode assemblages and diversity

**Fig 4:**
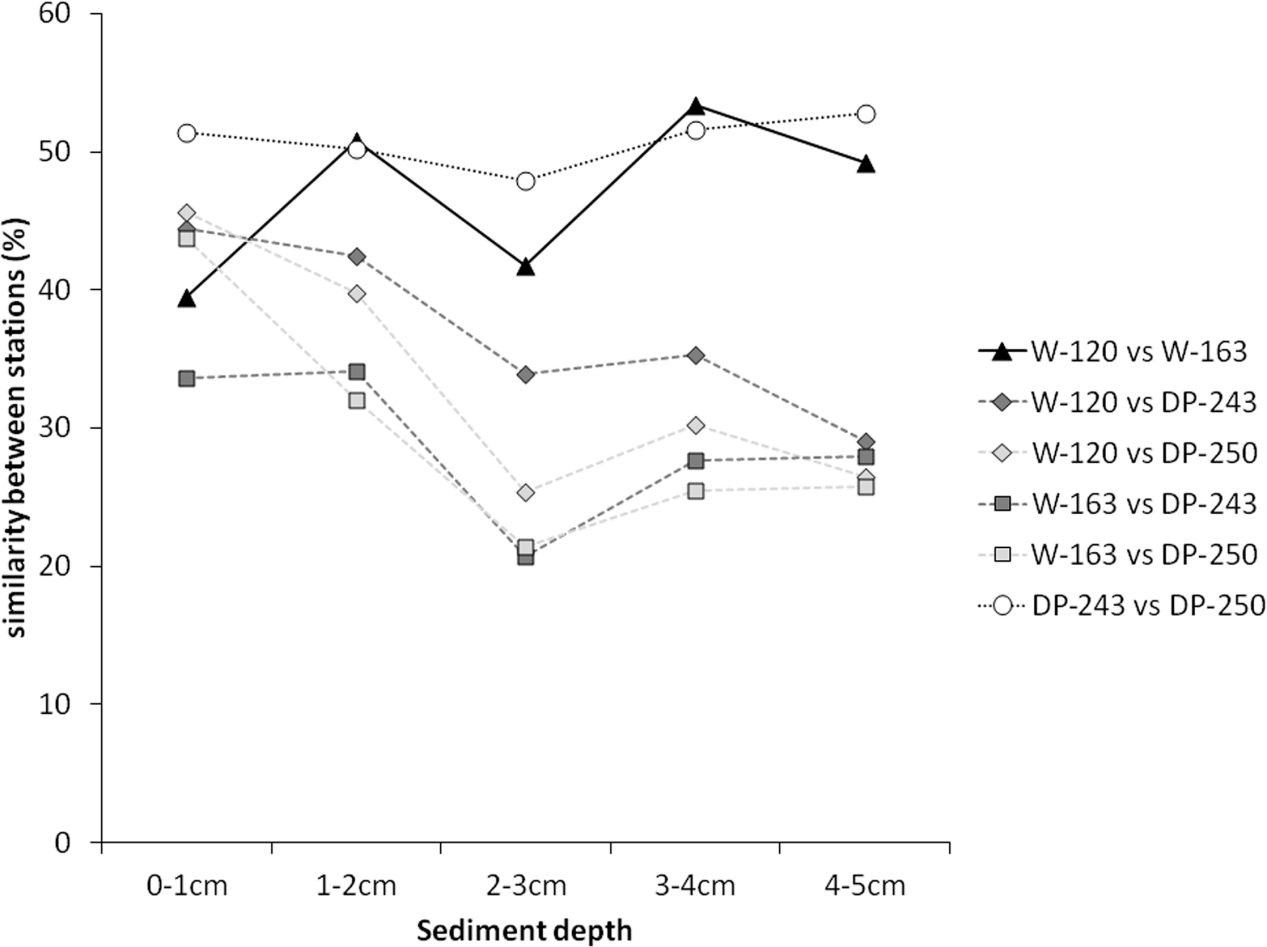
Visualisation of pairwise comparisons of the PERMANOVA interaction ‘station × layer’. Graph plots similarities of nematode assemblages between the different stations according to depth in the sediment.

Nematode assemblages were significantly different between areas (R = 0.84, p <0.05) and sediment layers (R = 0.62, p <0.05; two-way crossed ANOSIM), with differences increasing with sediment depth (ANOSIM pairwise comparisons). This was also revealed by PERMANOVA analysis (significant interaction effect of factors ‘station’ and ‘layer’; Table 4) and visualised in Fig 4. Analogously to the situation for the environmental setting (Fig 3), Drake Passage communities were more similar to each other for most sediment layers than those of the Weddell Sea. Within the same area, similarity between DP-243 and DP-250 remained relatively high throughout the sediment layers, while it varied with depth for stations W-120 and W-163 (Fig 4). When comparing similarities between stations across both sides, largest differences in communities were noted between station W-163 and both DP stations, while station W-120 was more similar to DP stations. For both W-163 and W-120, similarity with DP stations decreased when moving further down into the sediment, indicating that nematode assemblages were more divergent in deeper sediment layers. This was also revealed by ANOSIM pairwise tests. Pairwise comparisons for different sediment depths within the different stations rendered significant differences between both the first and the second centimetre with the deeper layers (mainly for stations W-120 and DP-250; detailed results not shown). PERMDISP of the interaction term ‘station × layer’ yielded a p-value of 0.654, meaning that dispersions are homogeneous in multivariate space. However, since within-group sample size is <5 (each station × layer combination has three replicates), this should be interpreted with care [74]. Horizontal (i.e. between stations) and vertical (between sediment depths) differences in community composition were confirmed by MDS and CLUSTER analyses (results not shown), which showed a segregation of upper cm-layers (0–1 cm and 1–2 cm) from the deeper layers for all stations.

**Table 4:** PERMANOVA of nematode assemblages in Weddell Sea and Drake Passage (main test results). Asterisks represent significant results.

Source	df	Pseudo-F	P(perm)	Unique perms
Area	0	No test		
Station	2	2.9196	0.0273*	8917
Layer	4	12.823	0.0001*	9917
Replicate (Station)	8	No test		
Station × Layer	8	1.4319	0.0086*	9803

Nematode assemblages in the Weddell Sea consisted of 74 genera belonging to 28 different families (mainly Comesomatidae, Chromadoridae and Monhysteridae), while 88 genera belonging to 29 families (mainly Xyalidae and Comesomatidae) were found in Drake Passage. Of these total numbers of genera, 54 were shared between the two locations (albeit in different abundance; e.g., *Microlaimus*, *Daptonema*, *Linhomoeus*), 20 occurred only in the Weddell Sea and 34 only in Drake Passage (e.g., *Dorylaimopsis*), yielding a total of 108 genera recognised in the samples. Average dissimilarity within and between regions is given in Table 5, together with the genera that contributed most to these dissimilarities (SIMPER). In terms of dominance, there were no highly dominant (relative abundance >25%) genera present in any of the four stations (maximum of 11–22%). Several genera occurred in relative abundance >1% (ranging from 15 genera in W-163 to 26 in DP-243), but there were many rare genera as well in all stations. A total of 43 genera were unique, meaning that they only occurred in one out of four stations, but none of them contributed a lot to total numbers.

**Table 5:** Dissimilarity (%) of nematode assemblages within and between areas (averaged over replicates and sediment depths) and first five genera contributing most to observed differences (SIMPER).

	Weddell Sea	Drake Passage
**Weddell Sea**	50.35% *Microlaimus Linhomoeus Daptonema Sabatieria Halalaimus*	
**Drake Passage**	67.75% *Microlaimus Linhomoeus Sabatieria Terschellingia Daptonema*	46.85% *Sabatieria Daptonema Dorylaimopsis Comesa Leptolaimus*

**Fig 5:**
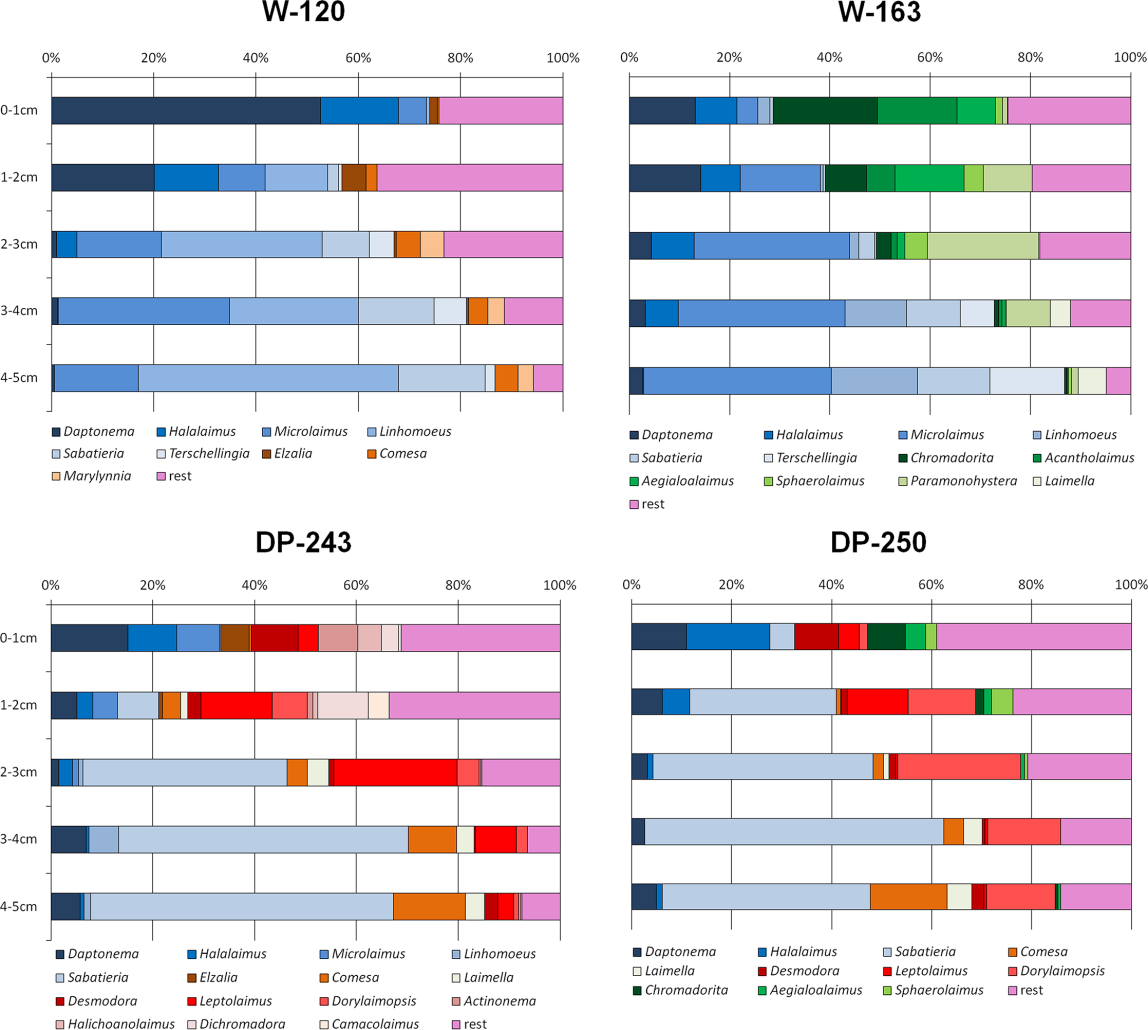
Vertical profiles of relative genus abundances for each station. Only genera with an abundance >4% in one of the layers were included, all others were grouped as “rest”. Where possible, we used the same colours for the same genera in all different plots.

Vertical profiles of nematode generic composition per station (Fig 5) showed that some genera were present throughout the samples (indicated in blue colours), while others occurred more specifically in one area (brown colours in W-120, green for W-163, and red for DP-243 and DP-250) or depth layer, or were shared between two locations. Community composition clearly changed with depth: genera *Daptonema* and *Halalaimus* were abundant in the first layers of both areas, but were replaced in the deeper layers by *Linhomoeus* and/or *Sabatieria*. As for the PCA and PERMANOVA results, W-120 and W-163 showed more variation in community composition among them than did DP-243 and DP-250.

**Fig 6:**
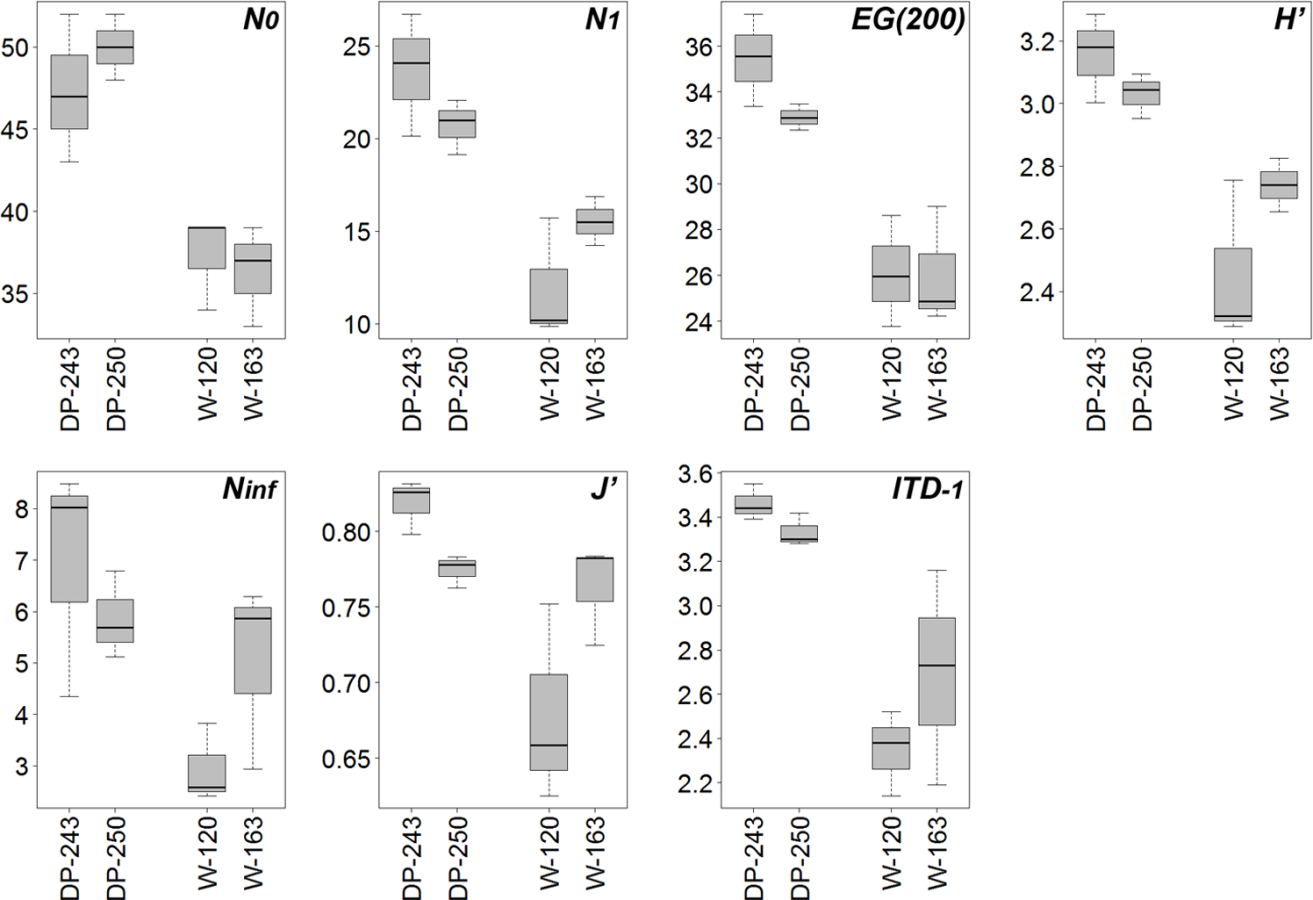
Box plots of the different diversity indices for the different stations. Boxes display median, first and third quartiles, minimum and maximum.

In terms of diversity, both sides of the Antarctic Peninsula showed differences, too. Average values of structural (diversity indices and evenness) and functional (ITD^-1^) diversity measures per station are listed in Table 6. Drake Passage stations exhibited highest values in general, for all diversity measures. One-way ANOVA for each index combined with post-hoc pairwise comparisons indicated that for most indices there were no significant differences between stations of the same area, but there were between stations of different areas (N_inf_ was never significantly different; Table 7, Fig 6). Both the observed number of genera N_0_ and the expected number of genera in a sample of 200 individuals, EG(200), were highest at stations DP-243 and DP-250; and lowest at W-163. For the other parameters (H’, N_1_) and evenness (N_inf_, J’), station W-120 had lowest values, while DP-243 remained highest. This means that communities at DP-243 were most diverse and had similar relative contributions of the various genera, whilst stations W-120, and to a lesser extent W-163, had lowest diversity with more variation in genus contributions to total abundance.

**Table 6:** Overview of structural diversity (*N*_*0*_, *EG(200)*, *H’* and *N*_*1*_) and evenness (*N*_*inf*_ and *J’*), as well as a functional diversity measure (*ITD* ^*-1*^) with their respective standard deviation for the nematode assemblages of the different stations calculated in PRIMER and averaged over three replicates.

	*N*_0_	*EG*(200)	*H*'	*N*_1_	*N*_inf_	*J*'	*ITD* ^*-1*^
**W-120**	37.33 (2.89)	26.12 (2.42)	2.46 (0.26)	11.94 (3.29)	2.94 (0.77)	0.68 (0.07)	2.35 (0.19)
**W-163**	36.33 (3.06)	26.02 (2.60)	2.74 (0.08)	15.52 (1.32)	5.03 (1.83)	0.76 (0.03)	2.69 (0.49)
**DP-243**	47.33 (4.51)	35.44 (2.01)	3.16 (0.14)	23.63 (3.32)	6.95 (2.27)	0.82 (0.02)	3.46 (0.08)
**DP-250**	50.00 (2.00)	32.89 (0.58)	3.03 (0.07)	20.73 (1.48)	5.86 (0.85)	0.77 (0.01)	3.33 (0.07)

**Table 7:** One-way ANOVA results for each index with their P-values.

Df		Sum Sq	Mean Sq	F-value	P-value (>F)
***N***_***0***_	3	432.250	144.080	13.722	**
Station					
Residuals	8	84.000	10.500		
***N***_***1***_	3	246.155	82.052	12.744	**
Station					
Residuals	8	51.508	6.439		
***EG(200)***	3	206.518	68.839	16.198	***
Station					
Residuals	8	33.999	4.250		
***H'(loge)***	3	0.879	0.293	11.694	**
Station					
Residuals	8	0.201	0.025		
***N***_***inf***_	3	25.909	8.636	3.522	Ns
Station					
Residuals	8	19.615	2.452		
***J'***	3	0.031	0.010	6.968	*
Station					
Residuals	8	0.012	0.001		
***ITD*** ^***-1***^	3				**
Station		2.510	0.837	11.718	
Residuals	8	0.571	0.071		

Significance codes

***<0.001

**<0.01

*<0.05, ns = not significant.

Also trophic diversity (ITD^-1^) was higher at DP-243 and DP-250, and differed significantly from Weddell Sea stations (except for DP-250 and W-163). The Weddell Sea stations had high relative contributions of feeding type 2A (epistratum feeders), represented by 44% in W-120 and 50% in W-163. Type 1B (non-selective deposit feeders) was second most abundant with percentages of 37 and 26%, respectively. In stations DP-243 and DP-250, there was a more even distribution among feeding types (except for type 2B), with relative contributions around 20–35% (1B had highest percentages in both stations).

## Discussion

Our study area coincides with the collision of true Antarctic (i.e. Weddell Sea Gyre) and oceanic (i.e. ACC) water masses, resulting in clear differences in temperature and surface primary production at a relatively small geographical distance. Bentho-pelagic coupling is responsible for the translation of these differences in surface-water processes to the seabed, leading to a distinct environmental setting for the benthos (cfr. pigment and organic matter content). Larger (mega-)benthic communities are known to track these differences as cold Weddell water turns around the peninsula tip and meets and mixes with ACC warm water [47,49]. Therefore, a similar change in nematode community structure was anticipated. In terms of abundance, results of this study are comparable to previous Antarctic observations (e.g., [31,79–80]), yet exceed those of other areas worldwide [81–82]. Higher nematode densities in the northwestern Weddell Sea compared to Drake Passage are mainly the result of high subsurface, rather than surface abundances (see Fig 2). Conversely, nematode genus richness is highest in Drake Passage stations. As hypothesised, nematode community composition varies depending on the region and is related to prevailing oceanographic and environmental conditions. Seasonal sea-ice retreat and subsequent enhanced food availability in the Weddell Sea at the time of sampling result in a community dominated by opportunistic genera (e.g., *Daptonema*, *Microlaimus*) able to benefit from deeper oxygen and food penetration (judging from their high numbers in subsurface layers; Fig 2). Conversely, open oceanic conditions and presumed low organic matter flux in Drake Passage triggers dominance of long motile nematodes such as *Sabatieria*, *Dorylaimopsis* and *Comesa*. These findings confirm the hypotheses that oceanic differences (i.e. temperature and water-column processes) between both east and west Antarctic Peninsula result in different nematode communities, mainly through indirect controls on food availability.

### Oceanography and primary productivity

Assessment of parameters in the water column confirmed that the stations in this study are influenced by different water masses. Deep cold Antarctic Bottom Water formation in the Weddell Sea is responsible for observed surface and bottom temperature differences between W and DP stations (almost -2°C vs. ~1°C, respectively). As this bottom water flows northward along the Weddell basin, it fuels thermohaline circulation and transports oxygen and nutrients on a global scale. Cold water combined with cold atmospheric conditions in the Weddell Sea area results in sea-ice cover present throughout most of the year, rendering primary production highly seasonal. However, upon annual sea-ice melt in austral summer, the meltwater enhances water-column stability and seeds regional phytoplankton (predominantly diatom-based) blooms in the Marginal Ice Zones (MIZ; [83–86]) and temporary polynyas near the continent [29]. This local and temporal enhancement of biogenic material [84,87] is further complemented by sea-ice algae released upon ice melt, which can account for up to 25% of total annual primary production in ice-covered waters [88]. Produced phytodetritus is transported rapidly through the water column, e.g., in the form of faecal pellets of zooplanktonic grazers (e.g., copepods and krill; [84,89]), resulting in seasonally high POC flux to the seafloor. Once at the seafloor, cold bottom-water temperatures lead to slow organic degradation rates and contribute to an accumulation of fresh organic matter or “foodbank” in the sediment [37,90] able to sustain a high meiobenthic standing stock throughout the year, even in deeper sediment layers [91,92]. Temperature thus plays a paramount role in food availability at both the surface and seafloor level.

Whereas sea-ice dynamics dictate food input in the eastern Antarctic Peninsula, continental shelves near the South Shetland Islands (region of DP-243 and DP-250) at the western side lie within the (usually) ice-free zones of the ACC [29]. The ACC abuts the continental shelves in this area, allowing Upper Circumpolar Deep Water (UCDW) to flood onto the shelf, principally through glacially carved canyons (as was observed from bathymetry onboard; [50,93]). This relatively warm UCDW (values of >1.5°C are not uncommon, but we encountered values around 1.2°C) is then mixed upward, introducing elevated concentrations of nutrients into the upper water column and allowing diatom-dominated phytoplankton assemblages to form subsurface chl *a* maxima above the pycnocline [94]. However, deep vertical mixing of ACC surface waters facilitates substantial recycling and consumption of phytodetritus by zooplankton already in the water column, accompanied by in-situ microbial degradation [29,95]. This typically results in a rather low carbon flux to the bottom [96–97]. The fraction that does reach the seafloor is then subject to lateral advection and resuspension by bottom currents, preventing sedimentation of finer fractions and fresh phytodetritus, and resulting in higher C:N values and lower pigment concentrations [98].

#### Weddell Sea dynamics and nematode abundance

High productivity and POC flux in Weddell Sea stations are confirmed by observed sediment pigment values but not reflected in surface water measurements and ocean colour data. Measured values for chl *a* in surface waters at the northwestern Weddell Sea tip (Table 2) are low compared to longer timescale averages [39] and reported values of >1.0 mg m^-3^ during phytoplankton blooms in areas similarly influenced by seasonal sea-ice retreat [99]. This observation presumably relates to timing of sampling, since the contribution of ice algae to primary production in the Southern Ocean generally peaks in December, a few weeks before maximum production rates are noted in open shelf waters [84,100]. Therefore, our snap-shot measurements most likely missed the actual blooming event while the satellite-based averages in Fig 1B failed to depict ephemeral sea-ice algal contribution. Despite low chlorophyll values in the water column, pigment values, the amount of fresh material and TOC content in Weddell Sea sediments are highest and remain high throughout the upper five centimetres. Encountered CPE values between 20 and 40 μg g^-1^ exceed those found in other areas worldwide (~1.4–6 μg g^-1^ at 200–800 m in the Central Mediterranean Sea; [101]) and in the Antarctic at similar times of the year (0.25–0.45 μg g^-1^ at a depth of ~400–550 m in the Ross Sea; [91]; ~0.5–1.6 μg g^-1^ at 750 m in the South Sandwich Trench; [102]). Pigment values indicate that the influence of a foodbank (see earlier) is more pronounced at station W-163, located deeper into the Weddell Sea, than at W-120 positioned at the tip of the peninsula, at the edge of the cold-water influence.

The combination of high fluxes of phytodetritus and cold bottom temperatures has resulted in higher meiofauna and nematode densities in Weddell Sea stations compared to Drake Passage, mainly in the subsurface. Congruence between sedimentary pigment values and nematode vertical profiles points to a drawdown of organic matter, either by biological activity and/or sedimentary processes in the form of increased mixing. In this respect, bioturbation by other benthic groups (mainly macro- and megafaunal burrowers) might play a key role in the oxygenation of deeper sediment layers and can lead to a transfer of organic matter to deeper horizons [103–105]. Although macrofauna organisms such as polychaetes and ophiuroids with the potential to rework upper sediment layers have been observed in the cores during sampling, no data on higher trophic level have been published so far for our stations. Therefore, the degree to which they might affect nematode communities and vertical distribution cannot be quantified. Alternatively, higher nematode abundance and deeper occurrence within seafloor sediments at W-120 and especially W-163 may (partly) arise from higher oxygen availability in this area compared to Drake Passage (due to oxygen-rich bottom water in the Weddell Sea; [106]).

In accordance with other findings (e.g., [58–59]), also here, high primary production and food availability lead to high numbers of benthic nematodes, which is indicative of strong bentho-pelagic coupling [60] and confirms the first hypothesis.

#### Drake Passage dynamics and nematode abundance

As opposed to Weddell Sea observations, high primary production in the water column, noticeable through intense coloration of filters, contrasts with lowest sediment pigment values and highest C:N ratios in Drake Passage stations. Chl *a* concentrations at the surface are within the range of satellite estimates at the time of sampling (see Fig 1C, Table 2), and at a broader temporal and geographical scope including the sampled area (1997–2006 averages of approximately 0.34–0.62 mg m^-3^ for the Southern Ocean, West Antarctic Peninsula and Weddell/Scotia Sea; [39]). In contrast, CPE content in Drake Passage sediments is much lower and mainly composed of phaeopigments, resulting in extremely low chl *a* concentrations (<0.1 μg g^-1^); even compared to other nearby shelf regions at the South Shetland Islands and Elephant Island (~0.6–0.8 μg g^-1^; [107]). Lower quantity and quality of phytodetritus in Drake Passage sediments probably result from water-column consumption and/or stronger bottom dynamics compared to the Weddell Sea, as discussed earlier. Contrary to Weddell sediments, where pigment values remain relatively high throughout the different depth horizons, their values rapidly decline in Drake Passage stations. Consequently, meiofauna and nematode density profiles follow a similar pattern of decrease with depth. This preference for surface sediment layers has been demonstrated in many studies (e.g., [108]), although occasionally also subsurface maxima have been observed [58,79,109].

### Nematode genus composition

Variation in oceanography and primary productivity at both sides of the peninsula has clearly resulted in differences in nematode abundance and vertical occurrence. Next to that, also nematode community composition and feeding mode differ depending on the area and depth in the sediment (see PERMANOVA results; Fig 5). Although most genera are not restricted to either Weddell or Drake Passage stations, their relative contributions vary for both areas and can be linked again to differences in environmental conditions.

In Weddell Sea sediments, surface layers (0–3 cm) contain high numbers of the genera *Daptonema* and *Halalaimus*, while *Linhomoeus*, *Sabatieria* and *Terschellingia* reside in deeper layers (3–5 cm). *Microlaimus* is present in considerable numbers throughout all layers. Similar depth ranges for these genera have been observed on different occasions (e.g., [60,110]). *Daptonema* and *Microlaimus* are comparably widespread in different oceans worldwide (e.g., [10,98,111]) and also *Halalaimus* is described as a eurytopic, cosmopolitan genus occurring in various types of sediments [110,112–113]. Because of its long thin shape, this latter genus can move easily through finer sediments (mainly associated with deep-sea habitats; [113–114]), such as the ones we observe in both Weddell Sea stations (Table 3). *Microlaimus* is considered a typical deep-sea genus, which can respond opportunistically to organic enrichment [110,114–115]. Therefore, the seasonally high flux of organic matter and freshness of deposited material in Weddell stations has lead to considerable numbers of this genus throughout all sediment depths, and explains why it is much less abundant in Drake Passage. Also *Terschellingia* is often present in organically enriched (mainly fine-grained) sediments [110,116].

In the Drake Passage stations, surface layers (0–1 cm) contain some of the abundant genera encountered in the Weddell Sea (*Daptonema*, *Halalaimus* and *Microlaimus*), but these are complemented by a variety of other genera, such as *Desmodora* (epistratum-feeder occurring mainly in surficial sediments; [10]) and *Leptolaimus*. Although *Desmodora* has previously been described as an opportunistic genus usually encountered in highly productive areas [112], here it is most likely associated with the coarser sediment in Drake Passage stations [60]. More strikingly, compared to Weddell stations, the genus *Sabatieria* becomes increasingly dominant in Drake Passage sediments, where it also occurs closer to the seafloor surface (already dominant from second centimetre onwards). Deeper down, it thrives in the company of *Leptolaimus*, *Dorylaimopsis* and *Comesa*. *Sabatieria* is known for its presence in sub- or anoxic conditions [110,117]. It tends to inhabit deeper sediment layers, mainly in association with the Redox Potential Discontinuity (RPD) layer [118–119], where its large body size and higher mobility allow it to move upward in the sediment to access oxygen and food in the upper layers [118]. Also *Leptolaimus* has been found in reduced deep-sea conditions (e.g., [120]). The presence of these genera might therefore indicate hypoxic conditions in Drake Passage stations although there were no clear visual signs (i.e. dark coloration of sediments, pronounced smell) of oxygen depletion observed deeper down in the sediment cores (unfortunately, precise oxygen profiles are lacking for all stations). Similarly as for *Sabatieria*, also *Dorylaimopsis* and *Comesa* have a long, slender body, which facilitates movement in the sediment. Well-oxygenated sediments at the Weddell side of the peninsula (see earlier) may explain why these genera are less abundant there.

In terms of dominant feeding mode, Drake Passage stations have more deposit-feeders (i.e. guilds 1A and 1B, together accounting for approximately 60% of total community) compared to dominance of epistratum-feeders (2A; approx. 50%) in the Weddell stations. Both epistratum- and deposit-feeders may use the same food sources, which are essentially limited to small particles (e.g., fungi, bacteria and unicellular microalgae; [76,121–122]). In this case, higher relative contributions of deposit-feeding genera such as *Sabatieria* in Drake Passage may result from the lower quality of deposited material, since they ingest particles as a whole [121]. Next to the obvious relationship between feeding mode and the nature and size of food particles, also temperature is known to affect feeding characteristics within benthic nematodes [123–124]. Details at the genus level are, however, too scarce to draw specific conclusions for this study.

Coming back to the hypotheses at the beginning of this study, above-described results on nematode genus composition confirm that not only megabenthic, but also smaller-sized meiobenthic communities respond to different oceanographic regimes around the Antarctic Peninsula, although shifts are less pronounced and mainly directed vertically. Predicted further warming of surface waters and atmospheric temperatures in the Antarctic Peninsula region will inevitably affect associated biota [124], judging from the tight link between oceanic features, primary production and nematode assemblages.
